# Health-seeking behaviour, health service delivery and its perceived impact among stroke survivors in Sierra Leone: a longitudinal qualitative study embedded in the SISLE project

**DOI:** 10.1186/s12913-025-13836-w

**Published:** 2025-12-02

**Authors:** Mamadu Baldeh, Jessica O’Hara, Divya Parmar, Christopher McKevitt, Daniel Youkee, Gibrilla F. Deen, Dimbintsoa Rakotomalala Robinson, Jotham Johnson, Augustine Thomas Mambu Bayoh, Albert Sama, Catherine Sackley

**Affiliations:** 1 College of Medicine and Allied Health Sciences, University of Sierra Leone, Freetown, Sierra Leone; 2 Medical Research Council Unit, The Gambia at London School of Hygiene and Tropical Medicine, Banjul, The Gambia; 3 King’s Global Health Partnerships, School of Life Course and Population Sciences, King’s College London, London, UK; 4 Stroke Research Group, Neurology Division, Neuroscience Institute, University of Cape Town, Cape Town, South Africa; 5 Stroke Association Sierra Leone, Freetown, Sierra Leone; 6 Faculty of Medicine and Health Sciences, University of Nottingham, Nottingham, UK

**Keywords:** Stroke, Sierra Leone, Qualitative, Stroke survivors, Caregivers

## Abstract

**Background:**

Stroke is a leading cause of disability and mortality globally. Despite growing awareness of the stroke epidemic, there is limited understanding of the lived experiences and perspectives of stroke care within resource-constrained health systems. This study explored the experiences and perspectives of stroke survivors, primary caregivers and healthcare providers on stroke care in Sierra Leone.

**Methods:**

This qualitative descriptive study involved face-to-face semi-structured interviews with stroke survivors, informal caregivers, and healthcare providers between December 2020 and August 2021 in Sierra Leone. We purposively sampled participants to capture diverse experiences across the care continuum. Data were analysed thematically using a constant comparative approach and interpretative phenomenological analysis, triangulating perspectives across participant groups.

**Results:**

Five interconnected themes emerged and are grouped into three categories: (1) Health-seeking behaviour: delayed hospital presentation, financial burden and out-of-pocket costs; (2) Health service delivery: hospital care experiences, access to and continuity of physiotherapy and; (3) Perceived impact and recommendation: recommendations for systemic improvements. Stroke survivors and caregivers reported a lack of knowledge of stroke symptoms, considering traditional treatment or religious consultation as the first point of contact, resulted in considerable delay in seeking hospitalisation. Other major barriers were related to challenges in accessing physiotherapy services and the financial hardship associated with stroke care. The dependence of stroke survivors on their caregivers as a result of staffing shortages and frequent communication breakdowns was frequently reported during hospitalisation. National health policies on stroke care, dedicated stroke units, community physiotherapy programmes and funding support were recommended.

**Conclusion:**

Stroke care in Sierra Leone is limited by systemic barriers that include infrastructure, cost, and accessibility. To bridge these gaps, community-based stroke education, health insurance schemes, workforce stroke-care training, and organised stroke-care delivery are needed. These results offer actionable recommendations for improving stroke care and service delivery in resource-constrained settings.

**Supplementary Information:**

The online version contains supplementary material available at 10.1186/s12913-025-13836-w.

¶Equal Contribution.

## Background

Stroke heavily burdens low-and middle-income countries (LMICs), where there are still systematic gaps in care [1–3]. Globally, stroke has high incidence and death rates, and Sub-Saharan Africa (SSA) is among the highest-burden regions [4, 5]. Despite the limited available data, the stroke burden appears to be increasing in SSA [5–7].

In Sierra Leone, emerging evidence indicates a significant burden of cardiometabolic risk factors [8–11]. The Stroke in Sierra Leone (SISLE) register is a comprehensive, three-year prospective longitudinal study by King’s College London and the College of Medicine and Allied Health Sciences, University of Sierra Leone. Between 2018 and 2022, SISLE enrolled 986 patients with confirmed stroke, making it one of the largest prospective stroke registers in Africa [8]. The SISLE register provided critical epidemiological insights into stroke risk factors, patterns of care, and patient outcomes, highlighting the urgent need for targeted interventions across the stroke care continuum [12–17].

Due to the profound impact stroke has on caregivers and families, it is sometimes referred to as a “family disease” [18, 19]. Disability is common among stroke survivors, who require continual family support and long-term professional care [20]. Supporting stroke survivors who are unable to take care of themselves is most of the time the duty of informal caregivers, who are typically family members. While studies have demonstrated that primary caregiver support to stroke survivors can improve well-being, emotional and functional recovery, caregivers are forced to alter their daily routines or abandon income-generating activities, resulting in significant financial strain on themselves as well as the whole family [20–22]. Effective stroke care is also a systemic problem for healthcare providers in resource-constrained health systems. Staff shortages, inadequate infrastructure (including neuroimaging) and limited funding make it more challenging to manage stroke patients [7].

Building on the quantitative findings of the SISLE register, this qualitative study aims to explore the lived experiences and perspectives of stroke care in Sierra Leone. To date, no qualitative studies have explored the lived experiences of stroke survivors and their caregivers in Sierra Leone, leaving these perspectives largely underrepresented in the literature and policy making. While the SISLE register has generated valuable quantitative data on the epidemiology and outcomes of stroke, it provides a limited understanding of how survivors and caregivers navigate the healthcare system, cope with post-stroke challenges, and perceive the quality of care received. Capturing these lived experiences will inform the design of patient-centred interventions for stroke and other non-communicable diseases (NCDs). Specifically, this study addresses the knowledge gap by examining health-seeking behaviours, barriers to accessing stroke care, rehabilitation and recovery challenges, and the broader health systems needs of stroke survivors, caregivers and healthcare workers. By centring participant voices, this research provides evidence on the complex relationship of individual, familial, community, and systemic factors shaping stroke care in a resource-constrained setting.

## Methods

### Study design

Following the Consolidated Criteria for Reporting Qualitative Research (COREQ), this study employed a qualitative exploratory study design [23]. Semi-structured interview guides were developed to explore the experiences of stroke survivors, caregivers and healthcare providers. To guarantee relevance to the local context, the study team, stroke survivors, caregivers, and healthcare providers collaborated in the development of the guides. The interview guides for each participant group are provided in the supplemental file [S1-S3]. The study protocol has been published [24].

### Setting

Sierra Leone is a low-income country in West Africa with a constrained healthcare system. Specialist services are limited, and at the time of the study, there was only one practising neurologist and two cardiologists serving a national population of over 8 million people. The study recruited participants through the SISLE register at Connaught Hospital in Freetown. This tertiary institution accepts referrals across Sierra Leone and serves a catchment population of over 1.2 million. Five medical teams, headed by a consultant physician, managed all admitted patients in the facility’s general medical wards. At the time of the study, there was no dedicated stroke unit or functional neuroimaging services. Since elevator access was unavailable, patients were usually admitted to the medical wards using wheelchairs and transported to upper-floor wards via the staircases. A separate department managed rehabilitation services and was often only engaged at the point of discharge, rather than being integrated into the acute or subacute phases of stroke care.

### Sampling and participants

We purposively sampled participants to maximise diversity in demographics, stroke severity, caregiving experiences, and health professional roles. Stroke survivors were identified through SISLE follow-up assessments. Eligibility criteria included being aged ≥ 18 years, at least three months post-stroke discharge, and having a National Institutes of Health Stroke Scale (NIHSS) score between 1 and 14 (mild to moderate stroke severity). To ensure that participants had adequate time to transition from hospital to home and reflect on their recovery journey and care experiences, we interviewed only those who had been discharged for at least three months. Conducting interviews immediately post-discharge may hinder reflective thought, as individuals typically concentrate on acute recovery and adjusting to new functional limitations. Primary caregivers were eligible if they had provided direct support to a stroke survivor within the preceding three months, including during the admission. Healthcare providers were included if they had at least 12 months of experience actively delivering stroke care at Connaught Hospital.

We interviewed 49 participants: 10 healthcare providers, 16 caregivers, and 23 stroke survivors, including two survivor-wife-sibling/adult-child triads and five survivor-caregiver dyads. The sample size was determined by data saturation rather than by numerical criteria. The objective was to conduct interviews with multiple stroke survivors from the SISLE registry and their caregivers until thematic saturation was achieved. This method continued data collection until saturation was reached. Through iterative analysis, data saturation was established when no new themes surfaced following the 40th participant interview. Recruitment was extended to 49 individuals, including four stroke survivors, three carers, and two healthcare providers.

### Patient and public involvement

Public and patient participation were integral to the study design and manuscript writing. The Stroke Association of Sierra Leone (SASL), a survivor-led advocacy group founded in 2019 as part of the SISLE project, was involved at every stage of this study. The interview guides were developed with input from SASL members during workshops and pilot sessions. The questioning style was revised to meet cultural sensitivity and relevance, and the interview duration was adjusted for the stroke survivor group to avoid fatigue.

### Data collection procedures

Between December 2020 and August 2021, data collection was conducted by trained qualitative researchers. The research team had established a relationship with participants through monthly SASL meetings. Interviews were done in either Krio (local dialect) or English, based on participant preference at a mutually agreed-upon venue. The interviews lasted between 35 and 80 min. Nonverbal cues and contextual situations were recorded in field notes.

Since data collection was undertaken during the COVID-19 pandemic, some participants reported increased worry, and movement restrictions periodically delayed interviews. The pandemic’s strain on Sierra Leone’s health system may have indirectly exacerbated stroke care challenges, though this study did not explicitly explore COVID-19’s clinical impact [25, 26].

### Data analysis

Interviews conducted in Krio were translated and transcribed by trained bilingual research assistants, and the English interviews were transcribed verbatim by the same team, all under the oversight of a senior local researcher. All transcripts were anonymised and stored securely with restricted access to authorised SISLE members. Data analysis followed a six-step interpretative phenomenological analysis (IPA) [27] and comparative thematic analysis: (1) M.B., D.R.R. and J.O. immersed themselves in data familiarisation, (2) notes on language, concepts, and emotions were made, (3) patterns and key ideas were systematically coded as emergent themes, (4) codes were grouped into preliminary clusters and organised in tables, (5) steps 1–4 were repeated across transcripts, and (6) themes were compared across stroke survivors, caregivers, and healthcare providers to identify overarching superordinate themes. Stroke survivor and caregiver accounts from dyads and triads were analysed independently to avoid data overlap. Separate coding frameworks were applied to each participant’s transcript to preserve individual perspectives. During theme clustering, shared experiences were cross-validated against non-dyad participants to ensure themes reflected broader patterns. All interview transcripts were handled and evaluated using NVivo software (QSR International, Version 12), which facilitated coding, classification and retrieval.

Two researchers (M.B. and J.O.) led the primary coding, with two independent senior researchers (D.P. and C.M.) conducting cross-validation to ensure interpretive accuracy of the codebook [Supplementary S4 Tables].

### Ethics approval and consent to participate

The Sierra Leone Ethics and Scientific Review Committee (SLESRC, 08/12/2020) and the King’s College London Research Ethics Subcommittee (HR-20/21-21050) approved this study. Each participant received an information pamphlet that explained the study’s objectives, methods, and their rights, including the option to withdraw at any time. This study follows the principles described in the Declaration of Helsinki, ensuring that all participants’ rights, dignity, and confidentiality are protected. Written informed consent was obtained from all study participants; when written consent was not possible, informed consent was obtained through a witnessed verbal procedure. Emotional distress was anticipated as a potential risk; interviewers were trained to recognise distress signals (e.g., visible crying, acute anxiety or agitation) and provide immediate support, including offering breaks or referrals as needed. Participation was voluntary, and participants could skip questions or discontinue at any time without penalty.

### Quality and rigour

To strengthen trustworthiness, we adopted rigorous steps as outlined by Lincoln & Guba [28] and Kocaman [29] to ensure our findings are credible, transferable, dependable, and confirmable. We ensured credibility (Internal Validity) of our findings through data-source triangulation: collecting and analysing data from the three study participant groups (survivors, caregivers, and healthcare providers). Transferability is ensured by providing detailed descriptions of the study setting, participant characteristics, and the research team’s positionality, enabling readers to assess the relevance of our findings to their own contexts. Similarly, the description of the study’s sampling strategy and participant selection criteria will help readers determine the applicability of the study findings to similar populations and settings. We acknowledge that while these descriptions provide informed assessment, the ultimate judgment of transferability rests with the reader.

Multiple steps were employed to ensure the dependability of our findings. These included following a standardised interview guide for each participant group, trained interviewers and a structured, team-based analysis process. Two researchers independently coded the data following a six-step protocol and then discussed and resolved any discrepancies. All decisions were recorded in a detailed audit trail. Linking our interpretations to verbatim quotes from participants ensures confirmability. Each key theme included quotes grounded in the participants’ own words, constantly tying analysis to raw data excerpts. Our reporting also included descriptions of the participant characteristics and the local healthcare context.

### Reflexivity

The SISLE team comprised clinicians, public health researchers, and social scientists from Sierra Leone and the UK. While the SISLE project provided diagnostic and logistical support to patients, we acknowledge the possibility of biases in how we present the project’s achievements and challenges. To mitigate this, we incorporated insights from caregivers and healthcare providers outside of SISLE into our analysis. The cultural expertise of local researchers contributed to a more nuanced understanding of faith-based and traditional practices, helping to ensure they were accurately represented. Partnering with SASL greatly enhanced the study design’s relevance, yet it also raised the risk of amplifying advocacy priorities. Data triangulation and differentiating the recommendations made by participants from those shaped by the researchers’ input addressed this issue.

## Results

The characteristics of the 49 participants interviewed are presented in Tables 1, 2 and 3. The age of stroke survivors ranged from 29 to 72 years. Most survivors (70%) were married and functionally dependent; caregivers were predominantly female (62.5%). All caregivers had assumed full-time or part-time caregiving responsibilities without formal training. Healthcare providers with clinical experience ranged from one year (house officers) to over two decades (senior clinicians and physiotherapists). Their professional responsibilities encompassed acute inpatient stroke care, discharge planning, rehabilitation, and overseeing follow-up care.

**Table 1:** Characteristics of the stroke survivor group

	Age (years)	Sex	Stroke period (months)	Marital status	Stroke effects
S1	45	Male	4	Married	Dependent, mobility problem, fatigue and anxiety/depression
S2	55	Male	3	Married	Dependent, mobility problem, numbness and anxiety/depression
S3	60	Female	3	Widowed	Dependent, mobility problem, weakness
S4	65	Female	5	Married	Dependent, mobility problem, weakness
S5	50	Male	6	Married	Dependent, speech, mobility and concentration problems, sexual dysfunction, anxiety/depression
S6	62	Female	7	Widowed	Dependent
S7	72	Male	4	Married	Dependent, mobility problem, hip pain
S8	48	Male	7	Married	Dependent, mobility problem, fatigue and anxiety/depression
S9	66	Male	6	Married	Dependent, mobility problem, imbalance and anxiety/depression
S10	59	Male	2	Married	Dependent, fatigue
S11	47	Male	4	Married	Dependent, mobility and concentration problems, anxiety/depression
S12	56	Female	3	Single	Dependent, speech problems, mobility problems, fatigue and anxiety/depression
S13	29	Female	15	Single	Dependent, speech problem, mobility problem, sleeplessness and anxiety/depression
S14	47	Female	13	Married	Speech and minor mobility problems, weakness and anxiety/depression
S15	50	Female	3	Single	Dependent, mobility problem, fatigue and anxiety/depression
S16	63	Female	3	Widowed	Dependent
S17	51	Male	6	Married	Dependent, mobility and concentration problems, sexual dysfunction and anxiety/depression
S18	42	Female	6	Married	Dependent, mobility problem, fatigue
S19	61	Male	5	Married	Dependent, speech, mobility and concentration problems, anxiety/depression
S20	64	Male	4	Married	Dependent, mobility problem, weakness and anxiety/depression
S21	49	Male	5	Single	Dependent, speech and mobility problems, anxiety/depression
S22	56	Female	7	Married	Dependent, concentration problem, foot weakness
S23	62	Male	15	Married	Partial dependent, concentration and mobility problems, anxiety/depression

*S: Stroke survivor

**Table 2:** Characteristics of the caregiver group

Caregivers	Age (years)	Sex	Marital status	Relationship to survivor	Duration caring for stroke survivor (months)
C1	27	Female	Single	Daughter	6
C2	45	Male	Married	Uncle	4
C3	32	Female	Married	Daughter	12
C4	56	Female	Married	Wife	8
C5	32	Female	Single	Granddaughter	6
C6	30	Male	Single	Brother	7
C7	27	Female	Single	Daughter	6
C8	30	Female	Single	Sister	6
C9	27	Female	Single	Daughter	4
C10	54	Female	Married	Daughter	7
C11	32	Male	Single	Son	12
C12	35	Male	Married	Son-in-law	7
C13	38	Male	Married	Son-in-law	6
C14	41	Male	Single	Son	6
C15	56	Female	Single	Sister	13
C16	43	Female	Married	Wife	15

*C: Caregiver

**Table 3:** Characteristics of healthcare provider group

Healthcare worker	Sex	Profession	Role/Position	Years of practice
HCW1	Male	Medical Doctor	House Officer	1+
HCW2	Male	Medical Doctor	Medical Officer	3+
HCW3	Female	Nurse	General ward	7
HCW4	Male	Medical Doctor	Registrar	11+
HCW5	Female	Nurse	Nurse Officer	10+
HCW6	Male	Medical Doctor	Senior Registrar	15+
HCW7	Female	Physiotherapist	Senior Therapist	22
HCW8	Male	Medical Doctor	Senior Physician	10+
HCW9	Female	Nurse	SCCHN/General Ward	10+
HCW10	Female	Nurse	Physiotherapy Unit Nurse	9+

*HCW: Healthcare worker

### Themes

**Fig. 1:**
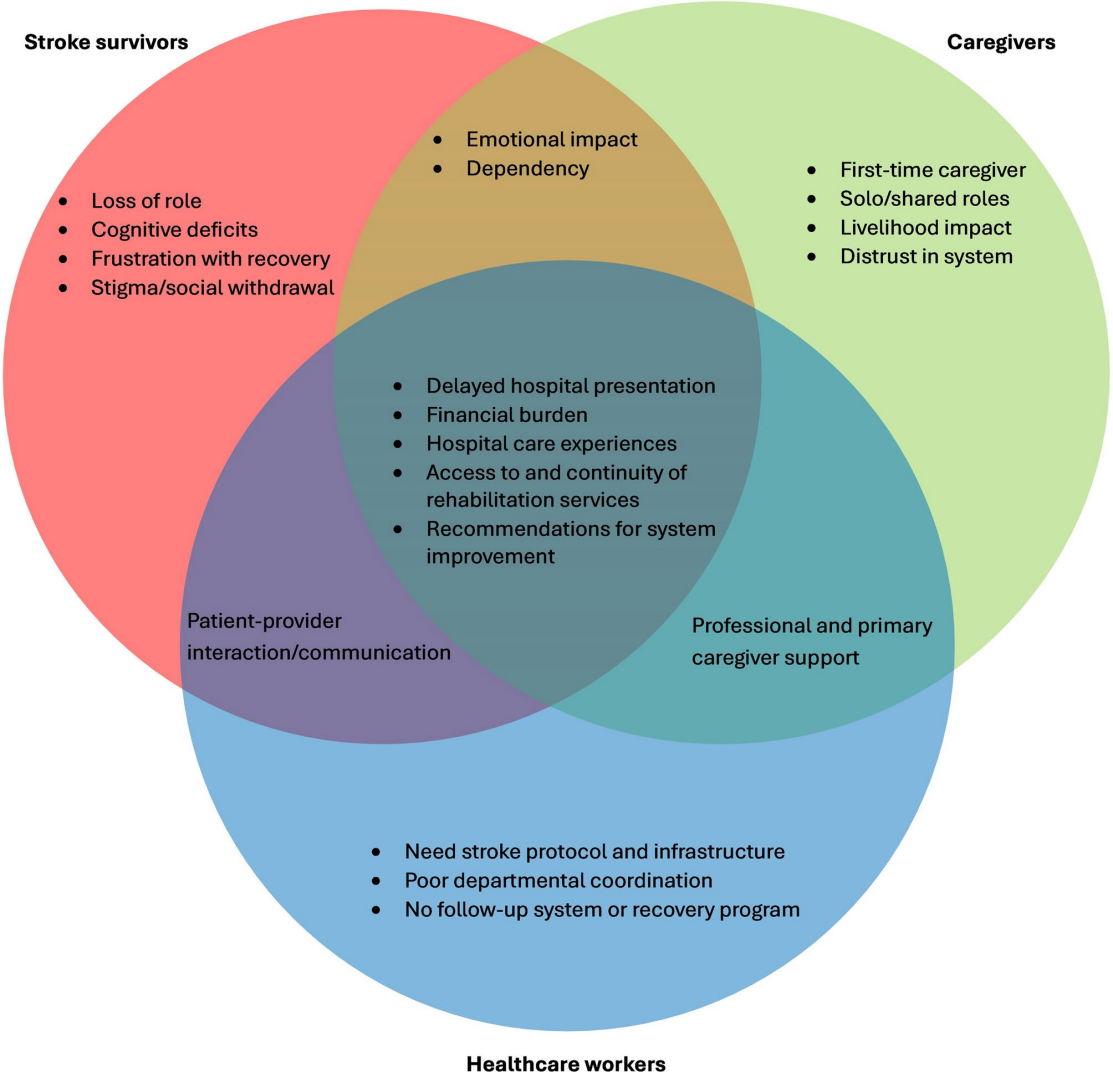
Thematic summary matrix. The red circle represents themes unique to stroke survivors, the green circle captures themes associated with caregivers, and the blue circle reflects themes reported by healthcare workers involved in stroke care. The central overlapping area illustrates themes shared across all three participant groups

Five broad themes were identified among all participant groups and grouped into three categories (Fig. 1): 1) Health-seeking behaviour (1a. Delayed hospital presentation, and 1b. Financial burden); 2) Health service delivery (2a & 2b. Hospital care experience, 2c. Discharge processes and post-hospital transitions, and 2d. Access to and continuity of physiotherapy services); and 3) Perceived impact and recommendation (3a. Improving Affordability and Access to Stroke Care, 3b. Expanding Community Education and Stroke Awareness, 3c. Strengthening Healthcare Workforce Training and Staffing, 3d. Developing Infrastructure for Stroke Care and 3e. Expanding Home-Based and Community Rehabilitation Services. Among the three thematic categories, ‘Perceived impact and recommendation ' emerged progressively, shaped by participants’ interactions with the healthcare system. Each theme presents quotes of the participants identified using the following abbreviations: S for stroke survivor, C for caregiver, and HCW for healthcare worker.

## Health-seeking behaviour

### Delayed hospital presentation

Across all participant groups, delayed hospital presentation was consistently identified as a major barrier to timely stroke care. Participants described critical gaps between the onset of symptoms and arrival time at a healthcare facility, primarily associated with limited awareness of stroke signs, misinterpretation of symptoms, and healthcare-seeking behaviour. Limited knowledge on presentation of stroke contributed to confusion among stroke survivors and caregivers regarding when to seek conventional medical help. Survivors failed to recognise the urgency of their symptoms, attributing initial weakness, speech difficulties, or facial asymmetry to transient illnesses such as malaria, fatigue, or spiritual affliction. Common terms used by participants to describe their initial presentation (translated verbatim) included ‘attack’, ‘tongue tangle’, and ‘bent mouth’ (facial drooping), as well as ‘heavy hand’ or ‘heavy foot’ (limb weakness), misinterpreting these signs to pursue spiritual, herbal, or home-based remedies. One survivor shared their initial misunderstanding of symptoms:

*At first*,* I just felt weak. I thought it was malaria or stress. It wasn’t until I couldn’t move my arm that my daughter called someone.* [S2, Male, 55 years]

A common theme was the reliance on faith-based healing pathways. Many survivors recounted reaching out to religious leaders or traditional healers right after their symptoms began. One participant noted:

*I called my pastor to inform him I had an attack*,* and he advised me to go to the church.* [S4, Female, 65 years]

Caregivers similarly described family tensions regarding care options, with some relatives preferring traditional or spiritual methods over medical treatments. Even when caregivers recognised the severity of the condition, prevailing family pressures and entrenched faith in non-medical solutions often delayed their decisions to seek formal healthcare services. Healthcare providers corroborated these accounts, describing medical pluralism with community preference for complementary therapies and limited referral pathway from primary healthcare to tertiary facilities. A medical officer observed:

*Many of them come late. They pass through two or three facilities or spend days at home or somewhere. By the time they get here*,* we are just managing complications.* [HCW2, Medical Officer]

Healthcare workers stressed that the absence of community-level stroke education exacerbates delays. A timely presentation within the stroke window period, the critical timeframe after symptom onset, is essential. Yet, delayed hospital presentation is influenced by a complex phenomenon of limited stroke awareness, sociocultural norms, and systemic barriers to healthcare access.

### Financial burden and out-of-pocket costs

Financial strain emerged as a cross-cutting barrier to accessing acute stroke care and long-term physiotherapy. Participants consistently described the catastrophic economic effects of stroke, with costs accumulating from transportation, medications, and recurring physiotherapy sessions. In Sierra Leone, where the health financing system is predominantly based on out-of-pocket payments, affordability is a decisive factor influencing care-seeking behaviour and treatment options. Public hospitals are considered an affordable care option; however, even within public facilities, families incur significant expenses. One stroke survivor explained the rationale for choosing Connaught (public) Hospital. Healthcare providers highlighted that even among individuals with private health insurance, payment for care remained overwhelmingly out-of-pocket. The absence of direct linkage between insurance providers and public health facilities meant that patients had to cover immediate expenses for medications and investigations before reimbursement. As a medical officer explained:

*Even when someone says they have insurance*,* it doesn’t help much here. They still pay first for tests*,* drugs*,* even consultations.* [HCW2, Medical Officer]

Indirect costs were a major reported challenge. Many caregivers described abandoning income-generating activities, depleting savings, or selling assets to afford the cumulative expenses of stroke care. When patients required prolonged hospitalisation, families had to budget for essentials such as food and sometimes lodging. For rural patients referred to urban centres like Freetown, the cost of accompanying family members placed additional pressure on the households. One caregiver recounted:

*Everything costs. We paid for gloves*,* drugs*,* even paper. I stopped my business to stay at the hospital and had no income for weeks.* [C16, Wife]

This also suggests that patients were asked to pay for even basic medical supplies, as the hospital was also under-funded. The financial burden stretched beyond inpatient care into physiotherapy, including expenses for medications, wheelchairs, physiotherapy sessions, and transportation to follow-up appointments. A nurse pointed out:

*The (stroke) project covers tests like ECG and CT (scan)*,* but they (patients) still need to buy their medications or pay for a wheelchair. If they can’t*,* it delays everything.* [HCW3, Nurse]

Healthcare workers observed that this financial strain frequently led to premature self-discharges or discontinuation of care. Families unable to meet ongoing costs sometimes opted to withdraw patients against medical advice and pursue traditional healing options or home-based management without professional oversight.

*Some just pack up and go home without telling us. They say they have no money left. Others take patients to herbalists because it’s cheaper.* [HCW9, Nurse].

Stroke survivors frequently voiced guilt and emotional distress over the financial burden their health issues placed on their families. They often felt like a financial liability, adding to their physical and mental pain:

*I feel like I’ve exhausted my family’s funds. Every day I see them struggling to find small money to buy my medicine.* [S11, Male, 47 years]

The absence of a formal financial protection mechanism for stroke care, whether through government health insurance, public subsidies, or social safety nets, was deeply felt.

## Health service delivery

Participants encountered a mix of supportive interactions and notable challenges in hospital-based stroke care. Their stories reveal the complex realities of providing and receiving care within a resource-constrained healthcare system.

### Positive experiences

Stroke survivors and their caregivers valued the assistance offered by the SISLE project. When they arrived at the facility, the SISLE team evaluated patients for stroke eligibility criteria. The SISLE project financed blood tests and neuroimaging required to confirm the stroke diagnosis, including the admission cost and transport for testing not available on-site. Although the SISLE team did not provide direct clinical care, they maintained close coordination with the primary medical team of each patient. Participants noted the SISLE team made concerted efforts to communicate their diagnosis, offer emotional support, and respond promptly to patient needs.

Healthcare providers observed improvements in delivering clinical care through the expedited diagnostic testing and prioritised neuroimaging, attributing this to the SISLE project.

*Before the (SISLE) project*,* stroke (diagnosis) would be delayed for days. Now we fast-track their scans*,* and that changes the way we manage them.* [HCW4, Medical Registrar]

The project’s payment for tests was reported as a huge financial relief for stroke patients.

### Negative experiences

Alongside the positive reports associated with the SISLE project, many participants described huge gaps in care delivery. Multiple survivors and caregivers shared their experiences of feeling ignored, experiencing breakdowns in communication, and receiving inadequate support while hospitalised. These challenges were frequently attributed to severe staff shortages, inconsistent nursing care, and inadequate infrastructure. One caregiver noted:

*Sometimes we waited the whole day without seeing a doctor. ‘The doctor is occupied*,*’ the nurses would inform us*,* yet we never received updates on the situation.* [C7, Daughter]

Family members reported feeling alone, particularly during evenings and weekends when the staff was scarce. Caregivers frequently undertook nursing responsibilities like feeding, cleaning, and helping with toileting, usually without any support or training.

*If you don’t have a family member there*,* you are in trouble. Relatives do most of the work because the nurses are too busy.* [S17, Male 51 years]

Healthcare workers acknowledged the strain imposed by staffing shortages and overwhelming patient loads. A frontline nurse summarised the situation:

*We want to give more attention*,* but with 20 patients and only two nurses*,* it’s hard. Caregivers have to step in.* [HCW9, Nurse]

Staff burnout and emotional fatigue were cited as recurrent challenges. The cumulative pressure of limited human resources and poor working conditions undermined their ability to deliver holistic care.

### Discharge processes and post-hospital transitions

Experiences during discharge further underscored differences in hospital care quality. Numerous caregivers and survivors noted being discharged without clear guidance on medication routines, rehabilitation plans, or follow-up appointments. Survivors frequently received vague instructions to “rest” or “keep taking medications,” lacking comprehensive information on secondary prevention measures or potential warning signs of complications.

*They just told us to go home and rest. No advice about exercise*,* no physiotherapy plan*,* nothing.* [C15, Sister]

Healthcare providers admitted that time constraints and patient load limited the counselling they could provide before discharge, especially when bed turnover was prioritised to accommodate new admissions.

*At times*,* we quickly discharge stroke patients due to bed shortages. Although we offer guidance*,* it often feels rushed*,* and we realise it falls short.* [HCW8, Senior Physician]

In general, the care provided in the hospital for stroke survivors varied significantly, largely influenced by staff-to-patient ratios and the dedication of individual staff members. Ongoing systemic issues have persisted in hindering the provision of quality stroke care.

### Access to and continuity of physiotherapy services

Participants repeatedly highlighted the essential role of physiotherapy in attaining functional independence following a stroke. Starting physiotherapy early was frequently linked to significant improvements in mobility, strength, and confidence among survivors. Caregivers reported feelings of hope and relief as they noticed even minor signs of physical recovery.

*She started walking again after two months of physio. We were so happy. It felt like a miracle after thinking she would never move again.* [C3, Daughter]

Physiotherapy staff echoed this sentiment, emphasising that early and consistent rehabilitation was a key determinant of good outcomes. Healthcare professionals noted that patients who participated in physiotherapy shortly after admission achieved improved recovery and reduced long-term disability rates.

*If they start exercises early—even passive movements—they have a better chance of walking and using their hands again.* [HCW7, Physiotherapist]

However, ensuring the continuation of physiotherapy after hospital discharge has been difficult. The most common barriers cited included high transportation costs, long travel distances from rural areas to urban centres, and the complete absence of physiotherapy services in many districts. A stroke survivor from a rural area recounted:

*I live in Kabala (Northern Sierra Leone). “Physiotherapy is unavailable. We sought treatment in Freetown but had to return home because of the high accommodation costs.* [S19, Male, 61 years]

Although some public facilities like Connaught Hospital subsidised physiotherapy services, patients still incurred out-of-pocket expenses for assistive devices and medications. Caregivers described the financial burden of paying for physiotherapy packages, which became unsustainable for many families over time. The halt in physiotherapy sessions led to considerable emotional and psychological effects. Survivors who had shown improvement but then regressed due to discontinued therapy expressed feelings of despair, hopelessness, and guilt.

*When I started walking a little*,* I felt alive again. But when we stopped physio*,* I became worse. I just stayed in bed. I felt useless.* [S14, Female, 47 years]

Healthcare workers recounted that many patients discontinued physiotherapy prematurely after discharge. Survivors who made initial progress often experienced a decline into paralysis or contractures after skipping sessions. The lack of community-based rehabilitation programs or home physiotherapy services worsened the accessibility disparity.

## Perceived impact and recommendation

Participants from all groups expressed a shared vision for improving stroke care in Sierra Leone. Their recommendations centred on five themes: affordability and access, community education on stroke, strengthening training and staffing, expanding physiotherapy services, and investing in stroke-specific infrastructure.

### Improving affordability and access to stroke care

Participants underscored the critical need for financial protections to mitigate the huge out-of-pocket expenses linked to stroke care. Survivors and caregivers called for government subsidies or complete coverage of acute stroke management, physiotherapy, and essential medications. Financial hardship was seen as not only a barrier to accessing initial care but also a major cause of interrupted rehabilitation and poor long-term outcomes.

*If stroke care was free or even half-supported*,* so many more people would get help early.* [C13, Son-in-law]

Healthcare workers echoed the call for systemic financing reforms, noting that cost-related delays frequently undermined the potential benefits of hospital-based interventions. Ensuring universal, affordable stroke care is fundamental to any serious effort to reduce the burden of post-stroke disability and mortality.

### Expanding community education and stroke awareness

Participants emphasised the crucial role of stroke education in communities to promote early recognition of symptoms and encourage timely health-seeking behaviours. Participants recounted experiences where a lack of awareness resulted in harmful delays, as patients often consulted traditional healers or religious leaders before reaching out to health facilities.

*If people knew about stroke signs like a heavy mouth or heavy hand*,* they would not waste time with prayer or native medicines.* [C8, Sister]

Healthcare providers urged the implementation of culturally sensitive public education campaigns that utilise radio, community health workers, local leaders, and religious institutions to share information in easily understandable formats and regional languages.

*We need to take stroke education to the markets*,* the churches*,* the mosques and wherever people gather. Posters alone are not enough.* [HCW1, House Officer]

Participants also emphasised the advantages that improving community understanding of stroke as a medical emergency could lead to earlier hospital presentations and better recovery outcomes.

### Strengthening healthcare workforce training and staffing

A common recommendation is to improve healthcare providers training in stroke care. Caregivers noted that many healthcare providers seemed to lack familiarity with the specific needs of stroke patients, resulting in perceived deficiencies in patient support and communication.

*Let them train the nurses and doctors more on stroke. We need people who understand what patients go through* [C 9, Daughter]

Healthcare professionals raised concerns about the lack of specialised personnel, such as physiotherapists, occupational therapists, and speech therapists, was limiting the recovery services available for patients.

*We need more than good diagnostics. We need recovery services—speech therapy*,* occupational therapy*,* follow-up in the community.* [HCW 7, Physiotherapist]

Many healthcare providers support continuous professional development programs, workshops, and a national protocol for stroke care to provide consistent care across facilities.

### Developing infrastructure for stroke care

Participants showed strong support for setting up stroke units in major hospitals to facilitate coordinated, multidisciplinary care. Survivors and caregivers shared experiences where stroke patients were placed in general medical wards, leading to overwhelmed staff and overlooked stroke-specific needs.

*Let them build a stroke ward with real beds*,* staff who understand stroke*,* and clean toilets* [C10, Daughter]

Healthcare providers highlighted that a stroke unit, which integrates acute care, early rehabilitation, and discharge planning, could enhance clinical outcomes, reduce hospital stays, and improve service delivery.

*We need a real stroke unit where physio*,* nursing*,* and doctors work together from day one.* [HCW 8, Senior Physician]

There were also requests for essential infrastructural improvements, including reliable access to neuroimaging and suitable rehabilitation spaces equipped with physiotherapy tools.

### Expanding Home-Based and community rehabilitation services

Finally, participants recommended the establishment of mobile physiotherapy services and community-oriented follow-up programs to provide continuous care after discharge.

*After we left the hospital*,* there was nothing. No one came to check*,* no exercises. We just waited and prayed.* [S19, Male, 61 years]

Healthcare providers proposed establishing multidisciplinary community outreach teams capable of providing physiotherapy and secondary stroke prevention services at the household level, especially for patients in remote areas.

*We need teams that can go into the community*,* check on stroke patients*,* and keep their therapy going.* [HCW7, Physiotherapist]

Together, these recommendations informed how to deliver the pressing need for a holistic, system-wide approach to strengthening stroke care in resource-constrained settings.

## Discussion

This qualitative study explored the experiences and perspectives of stroke survivors, informal caregivers, and healthcare providers in Sierra Leone, generating insights into the multifaceted barriers and enablers along the stroke care continuum. Our findings highlight significant gaps across the domains of acute care access, financial protection, physiotherapy, and health system infrastructure, while also emphasising the urgent need for community education, workforce strengthening, and the development of stroke-specific services. These findings offer context-specific evidence to inform the design and implementation of stroke services in LMICs.

From previous studies conducted in sub-Saharan Africa, many survivors and their families delayed seeking biomedical care due to limited awareness of stroke symptoms, misinterpretation of early warning signs, and the influence of faith-based and traditional beliefs [30–32]. Several studies on explanatory models of health and medical pluralism in West Africa and Sierra Leone have highlighted that it is not uncommon for people to seek out various types of care for various reasons simultaneously [33–36]. Traditional healers and religious leaders have been successfully utilised to encourage hospital presentation in the context of the Ebola epidemic in Sierra Leone [37–40]. While medical pluralism is important for community health initiatives, it may also reflect scepticism toward conventional medical care.

Financial hardship was a common theme across all participant groups. Stroke care in Sierra Leone is predominantly financed through out-of-pocket payments, which include costs of diagnostics, medications, physiotherapy, transportation, and medical supplies. Even where partial financial support was available through the SISLE project, indirect costs remained a major barrier to sustained care. Costs of stroke care were often catastrophic for families. These findings build upon earlier regional studies and underscore the significant financial burden linked to stroke management, as well as the ongoing disparities in access to physiotherapy and rehabilitation services [5, 41–43]. Financial vulnerability not only delayed initial care-seeking but also led to interruption of physiotherapy and compromised long-term recovery.

Participants’ experiences in hospitals were mixed, revealing both resilience and systemic challenges. While some shared positive encounters with some healthcare providers, others raised concerns over poor communication, feelings of abandonment, and uncoordinated discharges. Factors contributing to these varying experiences included staffing shortages, infrastructure problems, and a heavy dependence on caregivers. These insights highlight broader criticisms of health systems where limited human resources and fragmented service delivery may undermine care quality [44].

Physiotherapy was widely recognised as pivotal to stroke recovery, yet consistent access to rehabilitation services was severely constrained by location, financial, and infrastructural barriers. In rural areas, rehabilitation gaps illustrate the spatial inequities entrenched by infrastructural barriers and physiotherapy equipment. Disruptions in physiotherapy exacerbate functional decline and emotional despair, reinforcing a cycle of dependency and disability. This has been reported in LMICs, where rehabilitation services remain grossly unavailable despite the growing burden of disability [30, 45, 46]. Expanding community-based rehabilitation programmes, including mobile physiotherapy teams, task-sharing, and exploring digital solutions could bridge gaps in access, particularly for rural populations [47].

Participants’ recommendations converged around several system-wide reforms deemed to be actionable. Although the World Health Organisation (WHO) recommendations prioritise stroke care in LMICs, the implementation gap is more prominent due to limited funding [48]. Developing stroke-responsive health systems necessitates ongoing investment in infrastructure, human resources, financial reforms, and culturally appropriate community engagement strategies. This study underscores the significant psychological impacts of stroke on survivors and caregivers, particularly the survivors’ guilt from financial dependency and the stress caregivers experience. While rehabilitation services prioritise physical recovery, emotional and psychosocial needs are often overlooked. This gap exemplifies the broader systemic neglect of mental health present in many LMICs [49–51]. Incorporating low-cost, task-sharing mental health interventions into rehabilitation programs could help bridge this gap and improve quality of life.

These findings are framed within global health policy priorities. The call for financial protection and equitable access to essential care aligns closely with the goals of Universal Health Coverage (UHC), which seeks to ensure that all individuals receive needed health services without financial hardship [52]. In addition, our results reinforce the relevance of the WHO Package of Essential Noncommunicable Disease (PEN) Interventions, which advocates for integrating cost-effective and sustainable NCD prevention and rehabilitation services into primary healthcare [53]. Ultimately, this study links the lived experiences of stroke survivors to policy action, providing evidence to inform national and regional strategies for developing equitable, resilient stroke care systems in Sierra Leone and other resource-constrained settings. A strength of this study is its triangulation of perspectives from survivors, caregivers, and healthcare providers, providing a holistic understanding of experiences in stroke care. Additionally, although the study reported on the experiences of individuals who sought medical attention at a single tertiary hospital, the majority of participants reflected on their journey both before admission and after discharge.

A number of study limitations must be noted. The study’s representativeness may have been limited since stroke patients classified as severe (NIHSS > 15), and those who were faced with communication challenges, were excluded. This decision involves ethical and practical considerations. Patients who have suffered a severe stroke may exhibit various neurological deficits, including aphasia and cognitive impairment, which can hinder their capacity to give informed consent or engage in qualitative interviews. The exclusion of severe stroke cases may have led to an under-representation of the perspectives of the most functionally dependent survivors, whose experiences with post-stroke care, rehabilitation, and psychosocial support may differ significantly from those of individuals with milder conditions. However, to limit this selection bias, we included caregivers who have provided support to patients with severe stroke, accounting for the hospital stay period, follow-up services and psychosocial impact on the stroke survivors.

Furthermore, while the SISLE project at the hospital facilitated diagnostic testing, the scenario does not accurately represent standard stroke care in Sierra Leone. Nevertheless, the findings of this study informed the development of the first nationally dedicated stroke unit and improvements in wheelchair accessibility. While this study does not assess the SISLE project, it provides essential insights into which aspects of the project were well-received by both patients and healthcare providers, guiding future stroke register studies in LMICs.

## Conclusions

This study provides an in-depth examination of the lived experiences of stroke care in the Sierra Leonean health system. Our findings proposed a phased implementation approach to addressing systemic challenges in fragile healthcare systems: a vision grounded in enhanced community education, financial protection, strengthened healthcare workforce training, expanded rehabilitation services, and the establishment of organised stroke care. These solutions, if implemented, have the potential not only to transform stroke outcomes but also to catalyse broader gains in NCD care and health system strengthening.

## Supplementary Information

Below is the link to the electronic supplementary material.

Supplementary Material 1

Supplementary Material 2

Supplementary Material 3

Supplementary Material 4

## Data Availability

Data are available upon reasonable request from the principal investigator of the SISLE project or the corresponding author, subject to evaluation and approval.

## Abbreviations

COREQ: Consolidated Criteria for Reporting Qualitative Research
HCW: Healthcare Workers
HIV/AIDS: Human Immunodeficiency Virus / Acquired Immunodeficiency Syndrome
IPA: Interpretative phenomenological analysis
LMICs: Low-and-Middle-Income Countries
NCDs: Non-Communicable Diseases
NIHSS: National Institute of Health Stroke Scale
SASL: Stroke Association of Sierra Leone
SISLE: Stroke in Sierra Leone Register
SLESRC: Sierra Leone Ethics and Scientific Review Committee
SSA: Sub-Saharan Africa
WHO: World Health Organisation
