# Supplementary material for: Health-seeking behaviour, health service delivery and its perceived impact among stroke survivors in Sierra Leone: a longitudinal qualitative study embedded in the SISLE project
Source: BMC Health Serv Res. 2025 Dec 2;26:25. doi: 10.1186/s12913-025-13836-w (PMC12777294; doi:10.1186/s12913-025-13836-w)
Supplement: Supplementary file 1 — Supplementary Material 1 [file 12913_2025_13836_MOESM1_ESM.docx]

***Topic guide (version 3.0): Semi-structured interview Guide (Stroke survivor)***

***Piloting***

This guide has already been piloted several times. The questions are designed to encourage participants to provide in-depth replies. The guide also contains follow-up questions to help participants clarify their comments. The follow-up questions are intended to determine how well participants understand the topic. Clarifying and recall queries assist in eliciting thorough replies and identifying information sources.

The guide is a flexible tool that provides a sound basis for exploring pertinent components associated with stroke and stroke care, such as presentation, health-seeking behaviour, health structure, financial implications, and social repercussions. It was adjusted in response to our preliminary and pilot sessions, which focused on the potential results and procedures pertinent to the final data collection.

***General Information***

***Introduction***

- The research team will be introduced, and the purpose of the interview will be clearly explained. This context is crucial for the interviewee to understand the relevance of their participation.
- Emphasise the significance of the interviewee's experience, as it is a crucial part of our research: We are keen to understand perspectives of stroke presentation, health-seeking behaviour, care received and, as your insights are invaluable to us.
- We understand the importance of your time and comfort during the interview process. Informed consent will be obtained from all participants before and at the start of recordings. This will be done in the appropriate and most convenient language, with details on steps, the voluntary nature of the interviews, and the right to withdraw. We will also ensure you are comfortable during the interview process, with periodic break periods and refreshments provided as needed.

We will remind all the participants of the confidentiality agreement and anonymity of the study.

| Date |  |
| --- | --- |
| Interview code |  |
| Start time |  |
| End time |  |
| Duration (minutes) |  |
| Location/Region |  |
| Sex of the participants |  |
| Profession/occupation |  |
| Relationship |  |
| Interviewer |  |

**Background/Icebreaker question**

*(Context on interviewee’s role and experience in living with Stroke)*

- Could you briefly tell us your age, profession before the Stroke, and describe your family?
- Were you aware of Stroke or knew someone who had one before your attack?
- Before your attack, were you aware of the risk factors or warning symptoms associated with Stroke?

**Could you please share your experience of the moment the stroke occurred?** We are interested in understanding your perspective on what happened, how you felt, and any details you remember about the situation leading up to the onset.

- Could you explain the events of the day you suffered a stroke attack?
- What were your pre-stroke signs?
- Who identified the issue first, and what action did they take?
- Did you visit other places before going to the hospital?
- What made you or your caregiver decide to go to Connaught Hospital?

**Can you describe your experience with the admission process to the hospital and the care you received?** Please include details about the process, the interactions with healthcare providers, and how you felt during your time in the hospital

- When you got to the hospital, what happened?
- Did you see a doctor right away, or did you have to wait?
- How much time did it take to be admitted to the observation and then to the ward?
- What was your impression of the care you received from medical staff when you were admitted?
- What was the duration of your hospital stay?
- Were you informed of your condition or what was going on by the medical team?
- Did you have any difficulties while you were there, such as communication difficulties, staff attitudes, or access to medication?
- Did you receive support from other (non-stroke) patients or non-relatives during your admission?
- How would you rate the treatment you got at Connaught Hospital?
- What memories do you have of your stay in the hospital?
- Describe some of the challenging events faced during admission

**Could you describe your experience with the discharge process at the hospital?**

- How was the discharge process? How did you get to know that you were about to be discharged?
- Were you given discharge instructions when leaving the hospital?
- Did the hospital staff tell you what to do when you got home?
- Did you get a referral for a follow-up appointment or physiotherapy?
- If so, did you go to follow-up appointments or physiotherapy? If not, what prevented you from doing so?

**Can you share your experience of life after the stroke?** We are interested in understanding how the stroke has impacted your daily life, your physical and emotional recovery, the challenges you face, and any changes in your relationships, work, or routine. How do you feel about your progress and the support you've received during this time

- Since returning home, what have been some of the most challenging things you've had to deal with?
- How has your capacity to work or take care of yourself been impacted by the stroke?
- What financial and emotional effects has it had on your family?
- Do you still work out at home or take any medications?
- Since your stroke, who has been assisting you at home?
- How have your friends, family, and community visited and supported you?

**Based on your experience, what recommendations would you have for improving care and support for stroke survivors, both during hospitalisation and after discharge?** Are there any specific changes or resources you believe would have helped you in your recovery

- If someone had just had a stroke, what guidance would you provide them?
- What adjustments do you think the community or hospital should make to assist stroke survivors better?
- Would you want to add anything further about your experience?

**Conclusion**

- Is there anything on this topic that I have not asked you about but that you think is important to tell me?
- Do you have any questions for me?

Thank you for all their inputs and time.
