# Supplementary material for: Health-seeking behaviour, health service delivery and its perceived impact among stroke survivors in Sierra Leone: a longitudinal qualitative study embedded in the SISLE project
Source: BMC Health Serv Res. 2025 Dec 2;26:25. doi: 10.1186/s12913-025-13836-w (PMC12777294; doi:10.1186/s12913-025-13836-w)
Supplement: Supplementary file 2 — Supplementary Material 2 [file 12913_2025_13836_MOESM2_ESM.docx]

***Topic guide (version 3.0): Semi-structured interview Guide (Informal caregivers)***

***Piloting***

This guide has already been piloted several times. The questions are designed to encourage participants to provide in-depth replies. The guide also contains follow-up questions to help participants clarify their comments. The follow-up questions are intended to determine how well participants understand the topic. Clarifying and recall queries assist in eliciting thorough replies and identifying information sources.

The guide is a flexible tool that provides a sound basis for exploring pertinent components associated with stroke and stroke care, such as presentation, health-seeking behaviour, health structure, financial implications, and social repercussions. It was adjusted in response to our preliminary and pilot sessions, which focused on the potential results and procedures pertinent to the final data collection.

***General Information***

***Introduction***

- The research team will be introduced, and the purpose of the interview will be clearly explained. This context is crucial for the interviewee to understand the relevance of their participation.
- Emphasise the significance of the interviewee's experience, as it is a crucial part of our research: We are keen to understand perspectives of stroke presentation, health-seeking behaviour, care received and, as your insights are invaluable to us.
- We understand the importance of your time and comfort during the interview process. Informed consent will be obtained from all participants before and at the start of recordings. This will be done in the appropriate and most convenient language, with details on steps, the voluntary nature of the interviews, and the right to withdraw. We will also ensure you are comfortable during the interview process, with periodic break periods and refreshments provided as needed.

We will remind all the participants of the confidentiality agreement and anonymity of the study.

| Date |  |
| --- | --- |
| Interview code |  |
| Start time |  |
| End time |  |
| Duration (minutes) |  |
| Location/Region |  |
| Sex of the participants |  |
| Profession/occupation |  |
| Relationship |  |
| Interviewer |  |

**Background/Icebreaker question**

*(Context on interviewee’s role and experience in working with a stroke survivor)*

- Please describe your relationship with the stroke survivor.
- Is this your first time providing care to a stroke survivor?
- How long have you been providing support to the stroke survivor?
- Before this current stroke attack, did you have any prior training or understanding regarding stroke?

**Could you please share your experience of the moment the stroke occurred?** We are interested in understanding your perspective on what happened, how you felt, and any details you remember about the situation leading up to the onset.

- Could you explain the first events leading up to the stroke?
- Who decided or made the decision on where to take the patient for care?
- Before seeking hospital care, did you attempt any traditional or religious visits/interventions?
- What challenges, including transportation, expense, or distance, did you face when bringing the patient to the hospital?

**Can you describe your experience with the admission process to the hospital and the care you received?** Please include details about the process, the interactions with healthcare providers, and how you felt during your time in the hospital.

- What was your experience of the admission process on arrival at the hospital?
- Did you experience delays in receiving care at the hospital? If so, what caused them?
- How did you feel about the care received at the hospital?
- Did you participate in providing care or support while the patient was admitted to the medical ward?
- What role did you perform while the patient was in the hospital?
- Did the healthcare provider assist you in your role?
- During your hospital stay with the patient, what were the general challenges that you faced?
- How much did you spend on treatment, transport, or medications during the hospital stay?

**Could you describe your experience with the discharge process at the hospital?**

- Did you get guidance or instructions on how to take care of the patient when you were discharged from the hospital?

**Could you describe your experience with providing home care?** Please include details about the transition from the hospital, the support you provided at home, and how you felt during this phase of your recovery

- When providing home care for the patient, what were the main obstacles you faced?
- Were you able to access any physiotherapy or follow-up care? Why or why not?
- How has this experience affected your personal life and family?

**Can you share your experience of the recovery process of a stroke survivor?** We are interested in understanding the challenges you faced, the support you provided, services availbale in you setting and your thoughts on your progress during this time.

- What is the patient's current state of recovery?
- What are the persistent challenges faced to stroke rehabilitation?
- Do you believe you have the skills or resources necessary to assist with rehabilitation at home?
- Has your role as a caregiver affected your emotional well-being or financial situation?
- Do you think there is stigma or discrimination toward stroke survivors in your community?
- What support do you think caregivers and stroke patients need most?

**Based on your experience, what recommendations would you have for improving care and support for stroke survivors, both during hospitalisation and after discharge?** Are there any specific changes or resources you believe would have helped you in your recovery

- How should stroke victims and their caregivers be treated in hospitals like Connaught, in your opinion?
- How can communities enhance early care and increase awareness of stroke?
- How would you advise others who are taking care of a stroke victim?

**Conclusion**

- Is there anything on this topic that I have not asked you about but that you think is important to tell me?
- Do you have any questions for me?

Thank you for all their inputs and time.
