# Supplementary material for: Health-seeking behaviour, health service delivery and its perceived impact among stroke survivors in Sierra Leone: a longitudinal qualitative study embedded in the SISLE project
Source: BMC Health Serv Res. 2025 Dec 2;26:25. doi: 10.1186/s12913-025-13836-w (PMC12777294; doi:10.1186/s12913-025-13836-w)
Supplement: Supplementary file 3 — Supplementary Material 3 [file 12913_2025_13836_MOESM3_ESM.docx]

***Topic guide (version 3.0): Semi-structured interview Guide (Healthcare workers)***

***Piloting***

This guide has already been piloted several times. The questions are designed to encourage participants to provide in-depth replies. The guide also contains follow-up questions to help participants clarify their comments. The follow-up questions are intended to determine how well participants understand the topic. Clarifying and recall queries assist in eliciting thorough replies and identifying information sources.

The guide is a flexible tool that provides a sound basis for exploring pertinent components associated with stroke and stroke care, such as presentation, health-seeking behaviour, health structure, financial implications, and social repercussions. It was adjusted in response to our preliminary and pilot sessions, which focused on the potential results and procedures pertinent to the final data collection.

***General Information***

***Introduction***

- The research team will be introduced, and the purpose of the interview will be clearly explained. This context is crucial for the interviewee to understand the relevance of their participation.
- Emphasise the significance of the interviewee's experience, as it is a crucial part of our research: We are keen to understand perspectives of stroke presentation, health-seeking behaviour, care received and, as your insights are invaluable to us.
- We understand the importance of your time and comfort during the interview process. Informed consent will be obtained from all participants before and at the start of recordings. This will be done in the appropriate and most convenient language, with details on steps, the voluntary nature of the interviews, and the right to withdraw. We will also ensure you are comfortable during the interview process, with periodic break periods and refreshments provided as needed.

We will remind all the participants of the confidentiality agreement and anonymity of the study.

| Date |  |
| --- | --- |
| Interview code |  |
| Start time |  |
| End time |  |
| Duration (minutes) |  |
| Location/Region |  |
| Sex of the participants |  |
| Profession/occupation |  |
| Relationship |  |
| Interviewer |  |

**Background/Icebreaker question**

*(Context on interviewee’s role and experience in providing care to stroke survivors)*

- Could you describe your professional background?
- What role/post do you now hold at this facility?
- How long have you been working in this role?
- Could you describe your regular daily routine in this facility?

**Can you describe your experience with the stroke admission process from a healthcare provider's perspective?** Please share details about the steps involved in admitting a stroke patient, the challenges you face, the communication with the patient and their family, and any resources or protocols that you rely on during this process.

- What is the typical admission process that stroke patients presenting to this facility have to follow?
- When admitting stroke patients, do you follow any specific criteria?
- What challenges do you face during the admission process?
- Based on your experience, what is responsible for delays in the hospitalisation of stroke patients?

**Can you describe your experience in providing care for stroke patients?** Please include details about the treatment protocols you follow, the challenges you face in delivering care, the support you receive from the healthcare team, and how you engage with patients and their families throughout the care process.

- Can you describe your experience with providing care to stroke patients?
- What role does the SISLE team at the Hospital play in supporting stroke care?
- Do you have any difficulties with resources, medication, or equipment while providing care?
- How well do the medical staff and other departments work together to provide stroke care?
- How do family members assist or support in the care of stroke patients?
- How helpful in your role are the caregivers when you're in the hospital?
- Have you encountered any disputes or difficulties with caregivers?

**Can you describe your experience with the discharge process and aftercare for stroke patients?** Please include details about how you ensure a smooth transition from the hospital to home, the instructions and support provided to patients and their families, and any follow-up care or resources available to aid in the patient’s recovery

- How do stroke patients usually get discharged?
- When patients get discharged, what type of instructions or information are provided to them?
- How is follow-up care provided to stroke survivors?
- Are there systems in place for community-based follow-up or home recovery support?
- What are some typical challenges that stroke victims encounter during rehabilitation?
- When does the physiotherapy service get involved in the patient care, and how accessible is it?

**Based on your experience in stroke care, what recommendations would you have for improving the care and support provided to stroke patients?**

- Are there any changes to the admission, treatment, discharge, or aftercare processes that you believe could improve patient outcomes or the efficiency of the care system?
- In your opinion, how could stroke care be improved in this facility and nationally?
- How could stroke physiotherapy services be more accessible and improved across Sierra Leone?
- What recommendations do you think are crucial to improving stroke care nationally?
- Based on your experience in managing stroke patients, do you have additional comments you would like to share?

**Conclusion**

- Is there anything on this topic that I have not asked you about but that you think is important to tell me?
- Do you have any questions for me?

Thank you for all their inputs and time.
